# Impact of the COVID-19 Pandemic on Pulmonary Hypertension Patients: Insights from the BNP-PL National Database

**DOI:** 10.3390/ijerph19148423

**Published:** 2022-07-10

**Authors:** Aleksandra Mamzer, Marcin Waligora, Grzegorz Kopec, Katarzyna Ptaszynska-Kopczynska, Marcin Kurzyna, Szymon Darocha, Michal Florczyk, Ewa Mroczek, Tatiana Mularek-Kubzdela, Anna Smukowska-Gorynia, Michal Wrotynski, Lukasz Chrzanowski, Olga Dzikowska-Diduch, Katarzyna Perzanowska-Brzeszkiewicz, Piotr Pruszczyk, Ilona Skoczylas, Ewa Lewicka, Piotr Blaszczak, Danuta Karasek, Beata Kusmierczyk-Droszcz, Katarzyna Mizia-Stec, Karol Kaminski, Wojciech Jachec, Malgorzata Peregud-Pogorzelska, Anna Doboszynska, Zbigniew Gasior, Michal Tomaszewski, Agnieszka Pawlak, Wieslawa Zablocka, Robert Ryczek, Katarzyna Widejko-Pietkiewicz, Jaroslaw D. Kasprzak

**Affiliations:** 1 1st Department of Cardiology, Bieganski Hospital, Medical University of Lodz, ul. Kniaziewicza 1/5, 91-347 Lodz, Poland; chrzanowski@ptkardio.pl; 2 Pulmonary Circulation Centre, Department of Cardiac and Vascular Diseases, Jagiellonian University Medical College, John Paul II Hospital in Krakowul, Pradnicka 80, 31-202 Krakow, Poland; marcin.waligoora@gmail.com (M.W.); grzegorzkrakow1@gmail.com (G.K.); 3 Department of Cardiology, Medical University of Bialystok, 15-276 Bialystok, Poland; kasia.ptaszynska@op.pl; 4 Department of Pulmonary Circulation, Thromboembolic Diseases and Cardiology, Centre of Postgraduate Medical Education, European Health Centre, 05-400 Otwock, Poland; marcin.kurzyna@ecz-otwock.pl (M.K.); szymon.darocha@ecz-otwock.pl (S.D.); michal.florczyk@ecz-otwock.pl (M.F.); 5 Institute of Heart Diseases, University Clinical Hospital Mikulicz Radecki in Wroclaw, ul. Borowska 213, 50-558 Wroclaw, Poland; mroczeke@wp.pl; 6 Department of Cardiology, Poznan University of Medical Sciences, 61-701 Poznan, Poland; tatianamularek@wp.pl (T.M.-K.); aniasmuk@wp.pl (A.S.-G.); michalwrotynski@gmail.com (M.W.); 7 Department of Internal Medicine and Cardiology, Medical University of Warsaw, 02-005 Warsaw, Poland; olga.dzikowska-diduch@wum.edu.pl (O.D.-D.); katarzyna.perzanowska-brzeszkiewicz@wum.edu.pl (K.P.-B.); piotr.pruszczyk@wum.edu.pl (P.P.); 8 3rd Department of Cardiology, Faculty of Medical Sciences in Zabrze, Medical University of Silesia, 41-800 Katowice, Poland; iskoczylas@tlen.pl; 9 Department of Cardiology and Electrotherapy, Medical University of Gdansk, 80-211 Gdansk, Poland; elew@gumed.edu.pl; 10 Department of Cardiology, Cardinal Wyszynski Hospital, 20-718 Lublin, Poland; blaszcz12345@interia.pl; 11 2nd Department of Cardiology, Faculty of Health Sciences, Collegium Medicum, Nicolaus Copernicus University, 85-168 Bydgoszcz, Poland; danuta.karasek@op.pl; 12 Department of Congenital Heart Disease, Institute of Cardiology, 04-628 Warsaw, Poland; bkusmier@gmail.com; 13 1st Department of Cardiology, School of Medicine in Katowice, Medical University of Silesia, 41-800 Katowice, Poland; kmiziastec@gmail.com; 14 Department of Population Medicine and Civilization Diseases Prevention, Medical University of Bialystok, 15-269 Bialystok, Poland; fizklin@wp.pl; 15 2nd Department of Cardiology, School of Medicine with Dentistry Division in Zabrze, Medical University of Silesia in Katowice, 41-800 Zabrze, Poland; wjachec@interia.pl; 16 Department of Cardiology, Pomeranian Medical University, 70-111 Szczecin, Poland; m1peregud@gmail.com; 17 Pulmonary Department, University of Warmia and Mazury, 10-357 Olsztyn, Poland; anna.doboszynska@wp.pl; 18 Department of Cardiology, School of Health Sciences, Medical University of Silesia in Katowice, 40-635 Katowice, Poland; zbgasior@gmail.com; 19 Department of Cardiology, Medical University of Lublin, 20-090 Lublin, Poland; mdtomaszewski@wp.pl; 20 Department of Invasive Cardiology, Polish Academy of Sciences, Mossakowski Medical Research Centre, Central Clinical Hospital of the Ministry of Interior, 02-507 Warsaw, Poland; a.pawlak1@wp.pl; 21 Department of Invasive Cardiology and Cardiology, Independent Public Provincial Complex Hospital in Szczecin, 71-455 Szczecin, Poland; wieslawa.zablocka@wp.pl; 22 Department of Cardiology and Internal Medicine, Military Institute of Medicine in Warsaw, 04-141 Warsaw, Poland; raryczek@gmail.com; 23 Department of Cardiology, Copper Health Center, 59-300 Lubin, Poland; kwidejko@wp.pl

**Keywords:** pulmonary hypertension, SARS-CoV-2, COVID-19, incidence, mortality, BNP-PL database

## Abstract

We aimed to evaluate the clinical course and impact of the SARS-CoV-2 pandemic on the rate of diagnosis and therapy in the complete Polish population of patients (pts) with pulmonary arterial hypertension (PAH-1134) and CTEPH (570 pts) treated within the National Health Fund program and reported in the national BNP-PL database. Updated records of 1704 BNP-PL pts collected between March and December 2020 were analyzed with regard to incidence, clinical course and mortality associated with COVID-19. Clinical characteristics of the infected pts and COVID-19 decedents were analyzed. The rates of new diagnoses and treatment intensification in this period were studied and collated to the proper intervals of the previous year. The incidence of COVID-19 was 3.8% (n = 65) (PAH, 4.1%; CTEPH, 3.2%). COVID-19-related mortality was 28% (18/65 pts). Those who died were substantially older and had a more advanced functional WHO class and more cardiovascular comorbidities (comorbidity score, 4.0 ± 2.1 vs. 2.7 ± 1.8; *p* = 0.01). During the pandemic, annualized new diagnoses of PH diminished by 25–30% as compared to 2019. A relevant increase in total mortality was also observed among the PH pts (9.7% vs. 5.9% pre-pandemic, *p* = 0.006), whereas escalation of specific PAH/CTEPH therapies occurred less frequently (14.7% vs. 21.6% pre-pandemic). The COVID-19 pandemic has affected the diagnosis and treatment of PH by decreasing the number of new diagnoses, escalating therapy and enhancing overall mortality. Pulmonary hypertension is a risk factor for worsened course of COVID-19 and elevated mortality.

## 1. Introduction

For nearly two years, the world has been struggling with the COVID-19 pandemic caused by SARS-CoV-2 coronavirus (severe acute respiratory syndrome coronavirus-2). The virus mainly affects the respiratory system but also presents with affinity to other organs, such as the endothelium, myocardiocytes, neural tissue and gut, wherever ACE-2 functional receptors enabling viral invasion are present. The clinical course of COVID-19 is diverse, from totally asymptomatic to severe respiratory distress syndrome requiring mechanical ventilation and associated with extreme mortality [1]. In clinical settings SARS-CoV-2 induces a significant prothrombotic effect due to heterogeneous mechanisms. COVID-19 coagulopathy combines the features of SIC (sepsis-induced coagulopathy), DIC (disseminated intravascular coagulation), TMA (thrombotic microangiopathy), HPS (hemophagocytic syndrome) and APS (antiphospholipid syndrome) [2,3]. As a result, pulmonary thrombosis and embolism often develop during infection, especially in patients (pts) admitted to hospital, and myocardial injury is common [3,4,5,6]. It was recognized early in the pandemic that comorbidities and cardiovascular risk factors accelerate the risk of infection and the unfavorable course of COVID-19 [4,7,8].

Pulmonary arterial hypertension (PAH) is a disease of the arterioles resulting in an increased resistance in pulmonary circulation and associated with elevated pressure in the pulmonary artery, with irreversible remodeling of the pulmonary arterial walls. Pulmonary hypertension is a heterogeneous illness, and only pts with PAH and chronic thromboembolic pulmonary hypertension (CTEPH), i.e., groups 1 and 4 according to WHO classification, are qualified for disease-specific treatment [9]. It is known that pts with PAH have a high risk of mortality during hospitalizations due to non-cardiovascular diseases [10,11,12]. Therefore, the combination of coronavirus infection and pre-existing pulmonary hypertension is a clinically difficult situation associated with a high risk of death, both due to the potentially severe course of COVID-19 and threatened right ventricular failure as a result of the worsening of pulmonary hypertension, e.g., due to hypoxia. In addition, in most of healthcare systems, the COVID-19 pandemic had a deep influence, leading to increased overall mortality due to hindered access to healthcare, diagnostics and treatment of diseases other than SARS-CoV-2 infection [10].

The aim of this study was to assess the leverage of the SARS-CoV-2 pandemic on the diagnostic and therapeutic process of PAH and CTEPH in Poland, including an analysis of the incidence and course of coronavirus infection in the population of patients with PH registered in the BNP-PL national database.

## 2. Methods

We analyzed documentation of the complete population of patients with PAH and CTEPH in Poland with diagnosis confirmed by right heart catheterization (RHC) and reported in the BNP-PL national database, which is regularly updated by all PAH/CTEPH centers. Thus, this analysis included 1704 pts (PAH, 1134; CTEPH, 570).

BNP-PL is the prime multicenter, prospective, complete registry of adult and pediatric patients with PAH and CTEPH created in central-eastern Europe. The structure of the BNP-PL registry (inclusion criteria and data collection) were previously described in detail [13,14,15]). Subjects from all 21 centers accredited by the National Health Fund (NHF) to treat PAH in adults in Poland are enrolled to the PAH adult arm of the registry [14]. All 16 centers dedicated by NHF to medical treatment of CTEPH in adults enroll subjects to the adult CTEPH arm of the registry [15]. The study protocol was revised and accepted by the Local Bioethical Committee (Nr KBE: OIL/KBL/27/2018).

## 3. Data Collection

The pts records in BNP-PL are updated annually. Following the rise of the COVID-19 pandemic, regional database supervisors were contacted to expand the information related to aspects of SARS-CoV-2 infection in the Polish adult PAH/CTEPH population. COVID-19 infection was required to be diagnosed with a genetic PCR test. Date, duration and outcomes of incident coronavirus infection were added to the database template. The information was provided by contributing centers using usual BNP-PL flow via web-based forms. Details of SARS-CoV-2 infection were collected by centers during follow-up visits related to the national drug program executed in person or via telemedical format. Thus, the analyzed data reflect COVID-19-related outcomes occurring in the period between March and December 2020.

We reviewed the frequency of coronavirus infections, the clinical severity of their course and the mortality, considering the specific therapies used. We compared the primary clinical characteristics of the group of infected and of deceased patients to the extant patients recorded in the BNP-PL database. In order to estimate the influence of the pandemic on PAH diagnostics, we compared the PAH/CTEPH incidence (new diagnosis rate) from March to December 2020 with the corresponding months of the former year. We also checked how the treatment profile of patients with PAH and CTEPH changed during the pandemic.

## 4. Statistical Analysis

For statistical comparison, the study cohort (adults from BNP-PL database) was divided into two groups: pts infected vs. non-infected with SARS-CoV-2. Furthermore, infected patients were analyzed according to the need for hospitalization. The subsets of survivors and non-survivors were compared with respect to the available clinical characteristics. Additional analysis was undertaken to compare COVID-19 in PAH vs. CTEPH subgroups. The most common comorbidities, occurring in about 10% or more of pts (listed in Table 1), were used for multivariate analysis and for estimation of comorbidity score, which was defined as the number of the following concomitant diseases: arterial hypertension, diabetes, coronary artery disease, hypothyroidism, chronic kidney disease, history of deep vein thrombosis and atrial fibrillation.

Constant variables were revealed as means (SD). Categorical variables were submitted as counts and percentages. Continuous variables were verified for normal distribution; for comparison between the two groups, we used the Student’s t test or the Mann–Whitney U test when suitable, and for categorical variables, we used the χ^2^ test with Yate’s correction as required. The significance was set at an alpha level of 0.05. Dell Statistica data analysis software (version 13, Dell, Texas, TX, USA, software.dell.com, accessed on 20 June 2022) was used for statistical analysis.

## 5. Results

### 5.1. COVID-19 Morbidity

**Figure 1:**
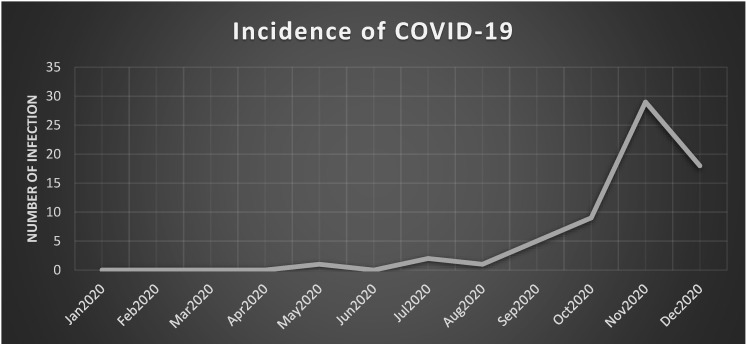
Incidence of COVID-19 among Polish PAH/CTEPH patients in 2020.

Between March and December 2020, the incidence of COVID-19 among the BNP-PL cohort was 3.8% (*n* = 65) and similar in the PAH (4.1%, *n*= 47) CTEPH (3.2%, *n* = 18) groups. The distribution of infections generally followed the waves of pandemic intensity in the overall population (Figure 1). Of the 65 pts with COVID-19, 25 (38%) required hospitalization due to symptomatic status and blood oxygen desaturation < 95%. Although the incidence of SARS-CoV-2 infections in the BNP-PL cohort was almost identical to that of the general Polish population (3.8% vs. 4.2%; *p* = 0.49) according to public domain data [16,17], the percentage of infected patients requiring hospitalization was much higher than the estimate for the general population (38% vs. 10–20%).

There was no difference in sex or age profile of PAH/CTEPH pts who contracted COVID-19 vs. the rest of the group (mean age, 55.7 ± 19.6 vs. 58.1 ± 17.4 years; *p* = 0.26). The prevalence of comorbidities such as hypertension, diabetes mellitus, coronary heart disease, hypothyroidism, chronic kidney disease or atrial fibrillation did not differ significantly between the pts infected with coronavirus and those not infected. Previous venous thromboembolism tended to be less frequent in the group of COVID-19 patients (9.2% vs. 18.4%; *p* = 0.06). The comorbidity score was similar between the two groups (3.0 ± 2.0 vs. 3.4 ± 2.2; *p* = 0.17) (Table 1).

### 5.2. COVID-19-Related Mortality

The mortality rate among PAH/CTEPH pts with COVID-19 was 28% (18/65), without statistical difference for hospitalized (34%; 11/32) vs. non-hospitalized pts (21%; 7/33)—remarkably higher than the 2.2% estimate for the general Polish population based on public domain data [16,17]. Those who died due to coronavirus disease were older (mean age, 68.4 ± 15.8 vs. 50.8 ± 18.8 years; *p* < 0.001) and had a more advanced functional WHO class (2.83 ± 07 vs. 2.47 ± 0.65; *p* = 0.05) and more cardiovascular comorbidities (comorbidity score, 4.0 ± 2.1 vs. 2.7 ± 1.8; *p* = 0.01). The most common comorbidity among patients who died during the COVID-19 infection was arterial hypertension (Table 2).

We separately analyzed PAH and CTEPH pts infected with SARS-CoV-2. In the PAH subgroup, the convalescents were younger than the decedents (mean age, 49.9 ± 18.8 vs. 69.3 ± 14.9 years; *p* = 0.003) and had a lower WHO functional class (2.5 ± 0.7 vs. 3.0 ± 0.7; *p* = 0,03) and a lower comorbidity score (2.3 ± 1.7 vs. 3.8 ± 2,2; *p* = 0.02) (Table 3). In the CTEPH subgroup, the survivors were not significantly different from those who died with respect to the analyzed variables (Table 3).

### 5.3. Impact of the Pandemic on Pulmonary Hypertension Care

The number of new diagnoses of pulmonary hypertension diminished by 25–30% during the pandemic period compared to the corresponding period in 2019 (total, 150 vs. 203; PAH, 90 vs. 123; CTEPH, 60 vs. 80). A significant enhancement in total mortality was also observed among the pulmonary hypertension pts included in the BNP-PL (9.7% vs. 5.9% pre-pandemic; *p* = 0.006). In addition, escalation of specific PAH/CTEPH therapies occurred less frequently (in 14.7% of pts vs. 21.6% pre-pandemic).

## 6. Discussion

The presented analysis of the BNP-PL database shows the effect of the COVID-19 pandemic on the diagnosis and treatment of pulmonary hypertension in Poland, as well as the course of this infection in patients with PAH and CTEPH, their prognosis and mortality, both general and related to SARS-CoV-2 infection. Our study is the first analysis of a complete national PAH and CTEPH registry focused on COVID-19 prevalence and its complications in the PAH and CTEPH populations.

It is clear that COVID-19 significantly affects healthcare systems and generates a considerable additional financial burden, which reveals its true syndemic aspects [10,18,19]. This aggravates the health situation in populations with predictable rises in future healthcare expenditure. Redirection of healthcare resources towards the pandemic reduces the intensity of primary and secondary prevention of cardiovascular disease and disrupts established pathways of care, such as PAH/CTEPH therapeutic programs in Poland.

Our study confirms that the rate of diagnosis of new PAH/CTEPH cases decreased significantly in Poland during the pandemic. The same observation was made in the USA [18]. This situation may be related to the repurposing of some accredited centers into temporary general COVID-19 centers, in addition to patients’ hesitancy to seek medical care or fear of presenting to a doctor or hospital, even with symptoms suggestive of pulmonary hypertension [20,21].

Guidelines for the diagnosis and treatment of patients with PAH/CTEPH recommend regular check-ups at a reference center and frequent diagnostic tests, including right-sided cardiac catheterization [9]. During the pandemic, the standard of care for these pts deteriorated significantly [22]. Most in-patient visits were replaced by telemedicine, and the number of invasive procedures was limited to urgent cases only. The number of laboratory tests performed in many centers as part of outpatient visits also decreased [10,23,24]. These factors explain the reduced rate of escalation of specific PAH /CTEPH therapies. We also observed an increased overall mortality in patients with PH, which may be related to both the limited access to healthcare caused by the SARS-CoV-2 pandemic and the fear of patients, who did not go to the hospital when clinical deterioration occurred.

In our population of PAH and CTEPH patients, the incidence of COVID-19 was comparable to that of the general population in Poland; similar observations were made in the USA, France, Germany and other European countries [18,19,24,25]. A potential susceptibility of PAH/CTEPH pts to pulmonary infection could be hypothetically balanced by an increased awareness of infection risk, leading to more social distancing and face mask use. A high level of anxiety associated with contacting healthcare providers at the time of the pandemic was also reported in pts with pulmonary hypertension [20,21].

The impact of PAH/CTEPH-specific therapies on infection risk was postulated shortly after the pandemic outbreak but does not seem to play an important protective role [26,27]. Contrary to suggestions that PAH may be linked to a lower risk of severe COVID-19 course [26,28], pts with PAH/CTEPH included in our study had a markedly higher mortality rate associated with COVID-19 than those uninfected and higher than that observed in the general population. Moreover, almost half of the patients with PH and COVID-19 required hospitalization—much more than in the general population, which indicates a more serious course of infection in people with PH. This observation, based on a complete national cohort, is in line with known clinical features of PAH/CTEPH patients and COVID-19 pathophysiology, encompassing hypoxia, inflammation and common myocardial injury.

## 7. Limitations

This study is limited by its retrospective nature and (typical of registries) might suffer from a fraction of missing data from the centers. Furthermore, we cannot exclude that some pts in the database may have experienced undiagnosed COVID-19 with absent or minimal symptoms or did not seek PCR confirmation with a nasopharyngeal swab. As the data were collected via telephone contact, adjudication was not always possible.

Our estimates of outcomes in PAH/CTEPH pts with COVID-19 should be interpreted with caution due to the low total number of patients with COVID-19 in our survey. Some indications related to COVID-19 in the general population (i.e., comorbidity profile) are not available, which limits the scope of feasible analysis. The presented data span the period preceding the introduction of vaccination in Poland in January 2021, and vaccination is likely to deeply impact the prevalence of COVID-19 among PAH/CTEPH pts. Finally, we could not ascertain the causal relationship between SARS-CoV-2 infection and out-of-hospital deaths.

## 8. Conclusions

Our study shows that the COVID-19 pandemic had a deep impact to the diagnostic and therapeutic routine of pulmonary hypertension care by decreasing the number of new diagnoses, hampering therapy escalation and increasing overall mortality in this population. This may be due, in part, to the conversion of some PAH and CTEPH centers into hospitals treating patients infected with SARS-CoV-2, as well as to patients’ fear of contacting healthcare professionals or consent to hospitalization in spite of aggravation. Pulmonary hypertension is clearly associated with remarkably increased mortality in COVID-19, as is the case for PAH and CTEPH.

## Figures and Tables

**Table 1:** Characteristics of patients with pulmonary hypertension (PAH and CTEPH) with or without COVID-19.

	Not Infected (*n* = 1639)	Infected with SARS-CoV-2 (*n* = 65)	*p*
**Age (years)**	58.1 ± 17.4	55.7 ± 19.6	0.26
**Females (*n*, %)**	1011 (61.7%)	42 (64.6%)	0.63
**WHO functional class**	2.5 ± 0.7	2.6 ± 0.7	0.18
**PAH (*n*)**	1087 (66.3%)	47 (72.3%)	0.32
**CTEPH (*n*)**	552 (33.7%)	18 (27.7%)	
**Eisenmenger syndrome (*n*)**	243 (14.8%)	7 (10.8%)	0.36
**Arterial hypertension (*n*, %)**	775 (48.1%)	26 (40%)	0.2
**Diabetes (*n*, %)**	264 (16.4%)	11 (16.9%)	0.88
**Coronary artery disease (*n*, %)**	249 (15.5%)	8 (12.3%)	0.49
**Hypothyroidism (*n*, %)**	289 (17.9%)	17 (26.2%)	0.09
**Chronic kidney disease (*n*, %)**	273 (16.9%)	8 (12.3%)	0.33
**History of DVT (*n*, %)**	296 (18.4%)	6 (9.2%)	0.06
**Atrial fibrillation (*n*, %)**	279 (17.3%)	12 (18.5%)	0.81
**Comorbidity score**	3.4 ± 2.2	3.0 ± 1.98	0.17

CTEPH—chronic thromboembolic pulmonary hypertension, PAH—pulmonary arterial hypertension, WHO—World Health Organization.

**Table 2:** Characteristics of pulmonary hypertension patients with COVID-19.

	Survivors (*n* = 47)	Died of COVID-19 (*n* = 18)	*p*
**Age (years)**	50.8 ± 18.8	68.4 ± 15.8	<0.001
**Females (*n*, %)**	32 (68.1%)	10 (55.6%)	0.35
**WHO functional class**	2.47 ± 0.65	2.83 ± 07	0.05
**PAH (*n*)/CTEPH (*n*)**	34/13	13/5	1
**Arterial hypertension (*n*, %)**	15 (31.9%)	11 (61.1%)	0.03
**Diabetes (*n*, %)**	7 (14.9%)	5 (27.8%)	0.23
**Coronary artery disease (*n*, %)**	5 (10.6%)	3 (16.7%)	0.51
**Hypothyroidism (*n*, %)**	11 (23.4%)	6 (33.3%)	0.42
**Chronic kidney disease (*n*, %)**	5 (10.6%)	3 (16.7%)	0.51
**History of DVT (*n*, %)**	3 (6.4%)	3 (16.7%)	0.2
**Atrial fibrillation (*n*, %)**	7 (14.9%)	5 (27.8%)	0.23
**Comorbidity score**	2.66 ± 1.8	4.0 ± 2.06	0.01

CTEPH—chronic thromboembolic pulmonary hypertension, DVT—deep vein thrombosis, PAH—pulmonary arterial hypertension, WHO—World Health Organization.

**Table 3:** Characteristics of patients with pulmonary arterial hypertension/chronic thromboembolic pulmonary hypertension and COVID-19.

	PAH			CTEPH		
	Survivors (*n* = 34)	Died of COVID-19 (*n* = 13)	*p*	Survivors (*n* = 13)	Died of COVID-19 (*n* = 5)	*p*
**Age (years)**	49.9 ± 18.8	69.3 ± 14.9	0.003	53 ± 19.5	66 ± 19.6	0.37
**Females (*n*, %)**	27 (79.4%)	8 (61.5%)	0.7	5 (37.5%)	2 (40%)	0.87
**WHO functional class**	2.5 ± 0.67	3.0 ± 0.71	0.03	2.4 ± 0.65	2.4 ± 0.55	0.96
**Arterial hypertension (*n*, %)**	11 (32.4%)	8 (61.5%)	0.07	4 (30.8%)	3 (60%)	0.27
**Diabetes (*n*, %)**	6 (17.7%)	3 (23.1%)	0.68	1 (7.7%)	2 (40%)	0.11
**Coronary artery disease (*n*, %)**	4 (11.8%)	2 (15.4%)	0.74	1 (7.7%)	1 (20%)	0.47
**Hypothyroidism (*n*, %)**	7 (20.6%)	6 (46.2%)	0.08	4 (30.7%)	0	0.17
**Chronic kidney disease (*n*, %)**	4 (11.8%)	2 (15.4%)	0.74	1 (7.7%)	1 (20%)	0.47
**History of DVT (*n*, %)**	0	1 (7.7%)	0.11	3 (23.1%)	2 (40%)	0.49
**Atrial fibrillation (*n*, %)**	5 (14.7%)	4 (30.1%)	0.22	2 (15.4%)	1 (20%)	0.82
**Comorbidity score**	2.3 ± 1.7	3.8 ± 2.2	0.02	3.6 ± 2	4.6 ± 1.8	0.35

CTEPH—chronic thromboembolic pulmonary hypertension, DVT—deep vein thrombosis, PAH—pulmonary arterial hypertension, WHO—World Health Organization.

## Data Availability

The data presented in this study are available on request from the corresponding author.
